# Supplementary material for: ASXL2 is essential for haematopoiesis and acts as a haploinsufficient tumour suppressor in leukemia
Source: Nat Commun. 2017 May 18;8:15429. doi: 10.1038/ncomms15429 (PMC5454368; doi:10.1038/ncomms15429)
Supplement: Supplementary Information — Supplementary Figures and Supplementary Table [file ncomms15429-s1.pdf]

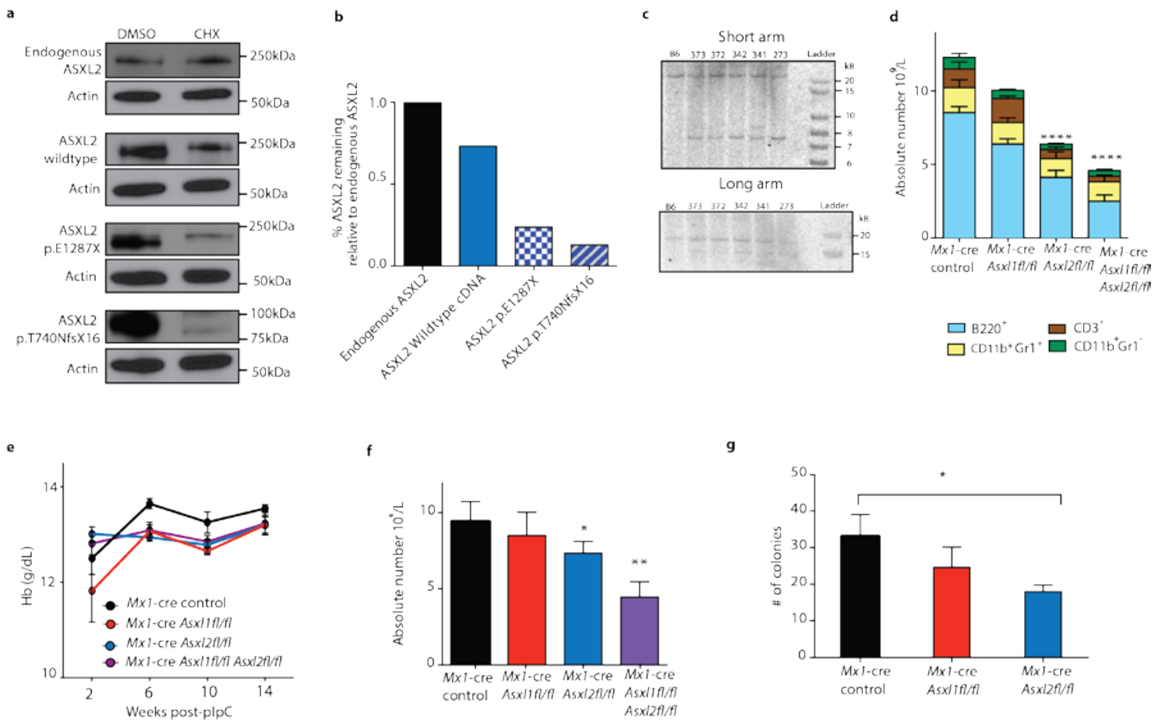

Supplementary Figure 1. ***ASXL2*** **mutations are loss-of-function mutations with a phenotype distinct from** ***ASXL1*** **loss.** (**a**) Transfection of 293T cells with wildtype ASXL2 cDNA or ASXL2 cDNAs bearing ASXL2 mutations (*ASXL2* c.3858_3859_insT (p.E1287X), c.2218dupA (p.T740NfsX16)) followed by treatment with DMSO or cycloheximide (CHX; 100ug/mL). Western blotting was performed after 12 hours of DMSO or CHX exposure using an anti-ASXL2 antibody. (**b**) Quantitation of ASXL2 protein expression relative to endogenous ASXL2 in CHX versus DMSO treated cells. (**c**) Verification of correct homologous recombination using Southern blots from targeted embryonic stem (ES) cells (C57BL/6 mice were the source of ES cells (labeled as "B6" in the figure; the clone number for each targeted ES cell clone is labeled above each lane of the gel). (**d**) Enumeration of B220$^+$, CD11b$^+$Gr1$^+$, CD11b$^+$Gr1$^-$ and CD3$^+$ cells in the peripheral blood of CD45.1 recipient mice 18 weeks following noncompetitive transplantation of bone marrow (BM) from CD45.2$^+$ *Mx1*-cre control, *Mx1*-cre *Asxl1fl/fl*, *Mx1*-cre *Asxl2fl/fl*, and *Mx1*-cre *Asxl1fl/fl Asxl2fl/fl* mice (n = 10 mice/genotype; pIpC was administered to recipient mice 4 weeks following transplantation). (**e**) Enumeration of hemoglobin (Hb) in CD45.1 recipient mice following noncompetitive transplantation of BM from CD45.2$^+$ *Mx1*-cre control, *Mx1*-cre *Asxl1fl/fl*, *Mx1-cre Asxl2fl/fl*, and *Mx1-cre Asxl1fl/fl Asxl2fl/fl* mice (n = 10 mice/genotype). (**f**) Flow-cytometric enumeration of BM megakaryocytes (CD41$^+$ CD71$^-$ cells) from the BM of mice with indicated genotypes (n=3 mice/genotype). (**g**) Enumeration of megakaryocyte progenitor colonies derived from lineage-negative Sca1$^+$ c-Kit$^+$ (LSK) cells from the indicated genotypes cultured in Mega-Cult media (n=3 mice/genotype). Error bars represent mean ± SD. **p*< 0.05, ***p*< 0.01, *****p*< 0.0001; *p*-values calculated by ordinary one-way ANOVA test.

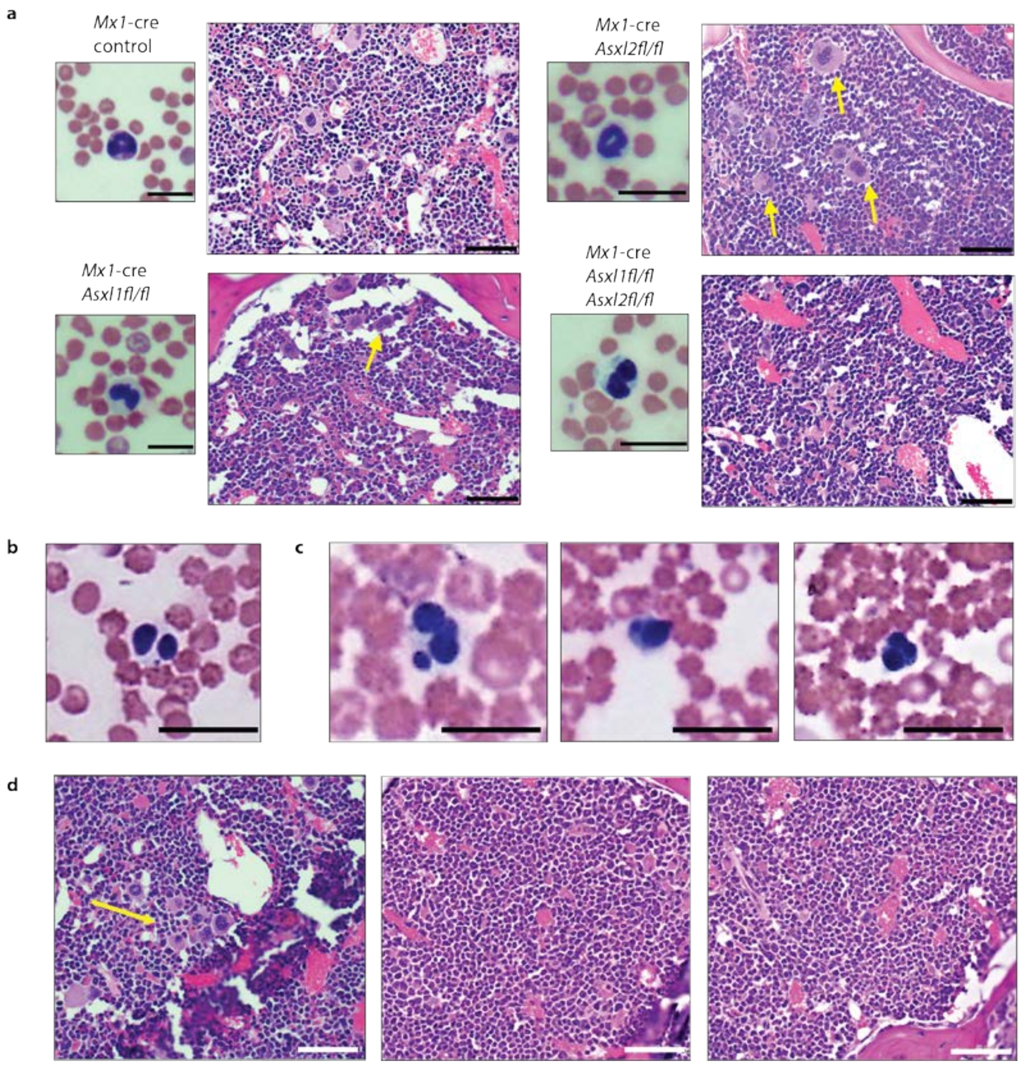

Supplementary Figure 2. Asxl2 loss is associated with defective megakaryocyte differentiation and myeloid dysplasia. (**a**) Representative images of peripheral blood (PB) show dysplastic neutrophils characterized by hypogranular cytoplasm in *Mx1*-cre *Asxl1fl/fl* and *Mx1*-cre *Asxl1fl/fl Asxl2fl/fl* with hyposegmented neutrophil nuclei noted as well in the *Mx1*-cre *Asxl1fl/fl* and *Mx1*-cre *Asxl1fl/fl Asxl2fl/fl* when compared to the *Mx1*-cre control (scale bars, 25µm, Wright-Giemsa stain) following noncompetitive transplantation. Bone marrow (BM) sections show dyplastic megakaryocytes characterized by separated nuclear lobules (yellow arrow) in *Mx1*-cre *Asxl1fl/fl*, abnormal megakaryocyte clustering (yellow arrows) in *Mx1*-cre *Asxl2fl/fl*, and small megakaryocytes with nuclear hypolobulation in *Mx1*-cre *Asxl1fl/fl Asxl2fl/fl* (scale bars, 50µm) following noncompetitive transplantation. **(b)** Hyposegmented neutrophils with hypogranular cytoplasm and **(c)** circulating, multinucleated erythroid progenitors in the PB of *Mx1*-cre *Asxl2fl/fl* mice (scale bar, 25µm). **(d)** Clustering of megakaryocytes (left image; yellow arrow) in the BM of *Mx1*-cre *Asxl2f/fl* mice despite overall decrease in megakaryocytes (as indicated by the middle and right panels; scale bar, 50µm).

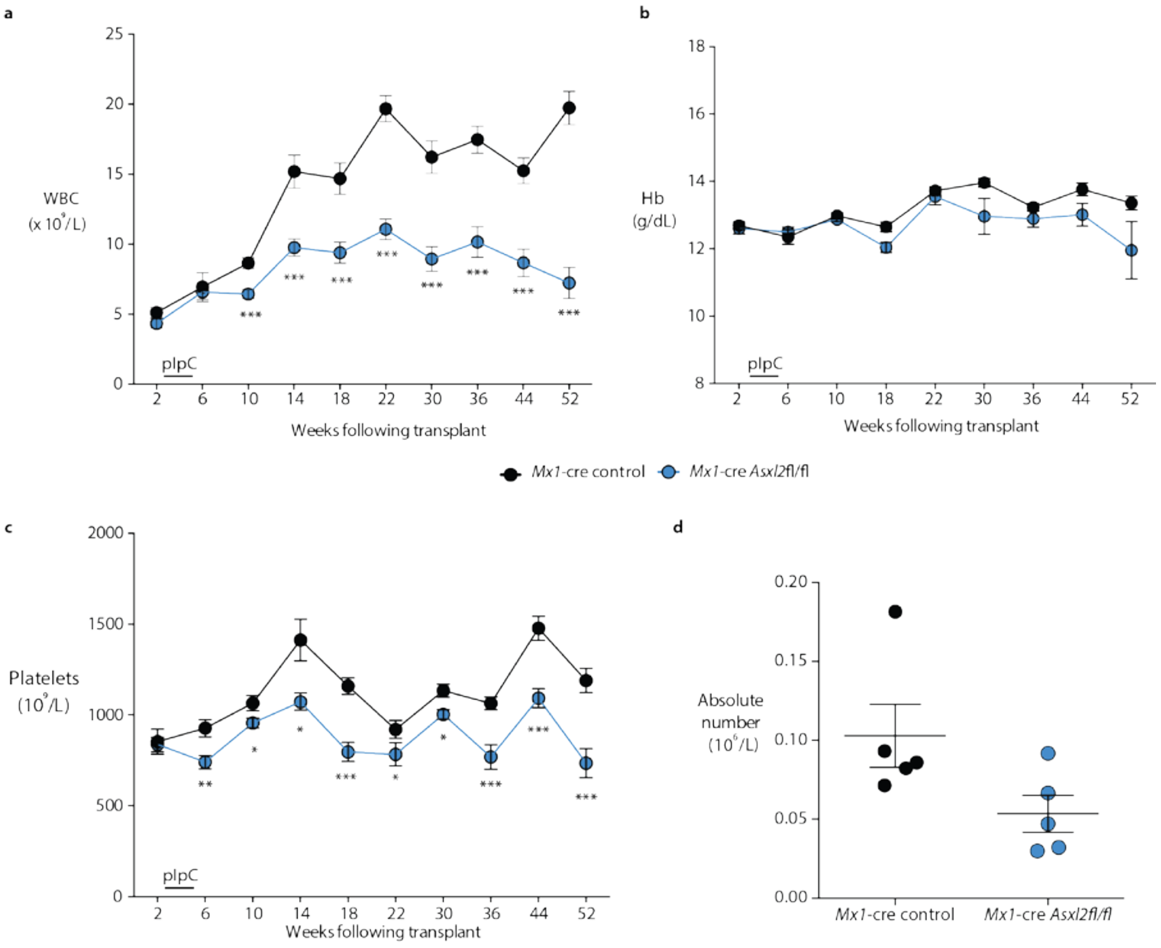

Supplementary Figure 3. Effects of *Asxl2* loss on hematopoiesis in the setting of noncompetitive transplantation. Peripheral blood **(a)** white blood cell (WBC), **(b)** hemoglobin, and **(c)** platelet counts of lethally irradiated CD45.1 recipient mice transplanted with CD45.2$^+$ *Mx1*-cre control or *Mx1*-cre *Asxl2fl/fl* bone marrow mononuclear cells (n=5 mice/genotype). pIpC was administered at 4 weeks post-transplantation and mice were followed for 52 weeks post-transplantation. **(d)** Absolute number of lineage$^-$ Sca1$^+$ c-Kit$^+$ bone marrow cells at 52 weeks post-transplantation from the mice shown in (a)-(c). Error bars represent mean ± SD. **p*< 0.05, ***p*< 0.01, *****p*< 0.0001; *p*-values calculated by ordinary one-way ANOVA test.

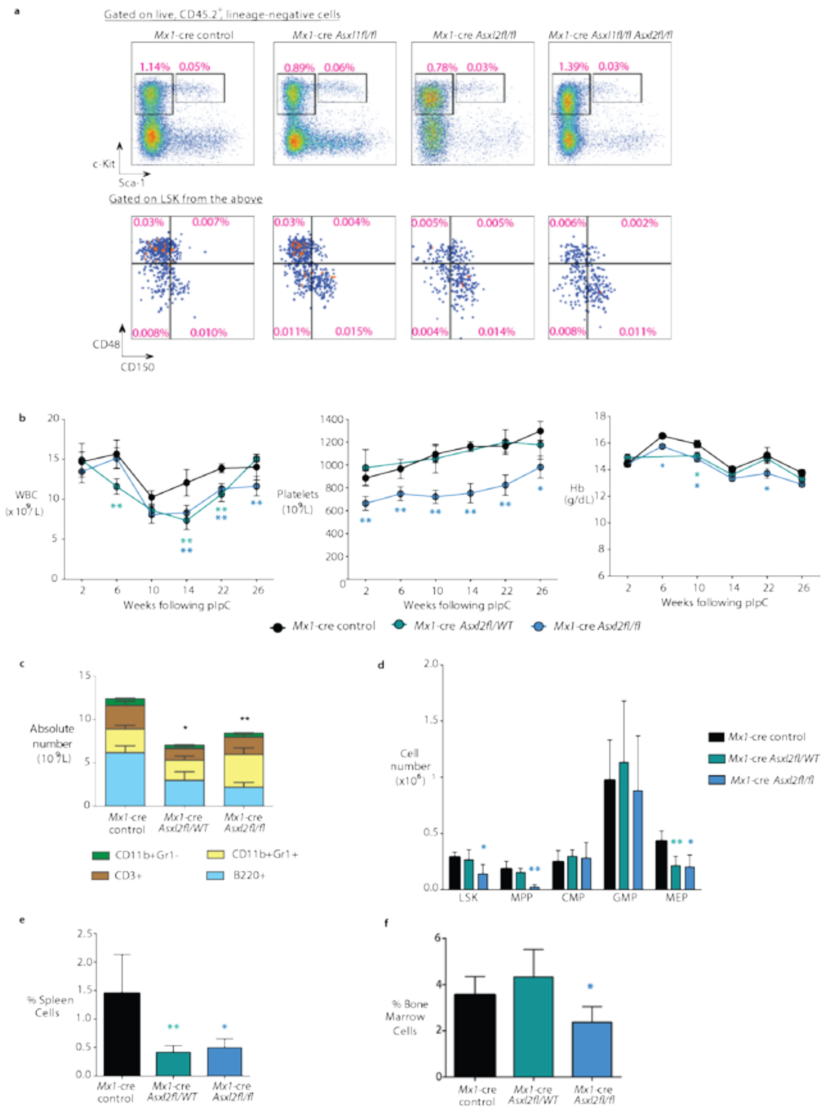

Supplementary Figure 4. Effects of Asxl2 loss on hematopoietic stem and progenitor cells in transplantation and non-transplantation settings. (**a**) Flow cytometric analysis of bone marrow (BM) hematopoietic stem and progenitor cells (HSPCs) following 16 weeks of pIpC administration to CD45.1 recipient mice transplanted with CD45.2 *Mx1*-cre control, *Mx1*-cre *Asxl1fl/fl, Mx1*-cre *Asxl2fl/fl,* and *Mx1-cre Asxl1fl/fl Asxl2fl/fl* BM cells (from Figure 2d). All percentages shown refer to percentages of live, CD45/2+, lineage-negative cells. **(b)** Peripheral blood (PB) white blood cell (WBC), platelet count, and hemoglobin in a cohort of primary, non-transplanted *Mx1*-cre control, *Mx1*-cre *Asxl2fl/WT,* and *Asxl2fl/fl* mice (n=6 mice/genotype in (b)-(f)) post-pIpC administration. All mice were treated with pIpC at 6 weeks of age. Absolute number of **(c)** PB mononuclear cell (MNC) types and **(d)** HSPCs from the mice shown in (b) at 22 weeks post-pIpC administration. Percentage of **(e)** $CD71^+$ $Ter119^-$ cells among spleen MNCs and **(f)** $CD41^+$ cells amongst BM MNCs from the mice in (b) at 22 weeks post-pIpC administration. Error bars represent mean ± SD. *p< 0.05, **p< 0.01, ****p< 0.0001; p-values calculated by ordinary one-way ANOVA test.

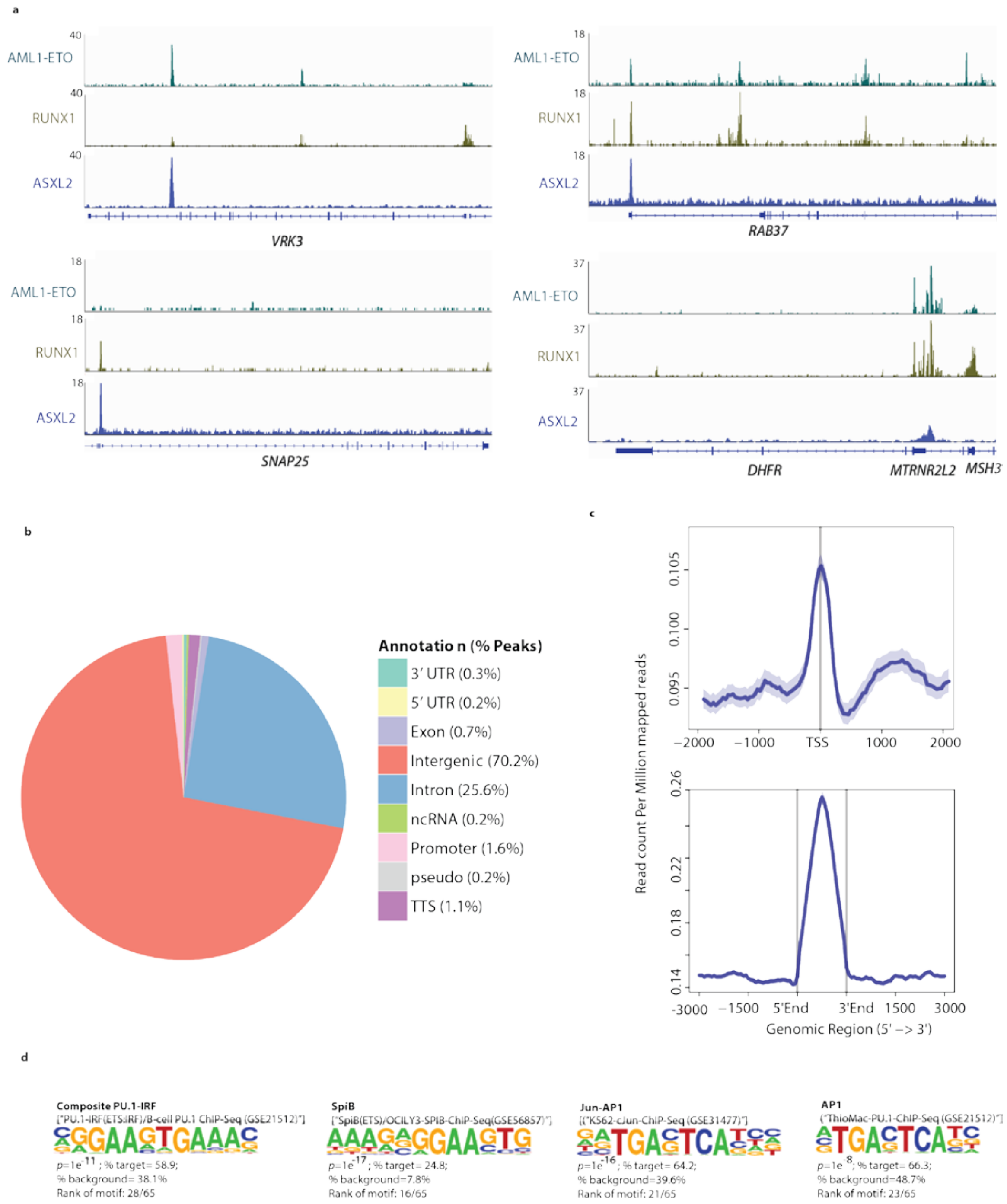

Supplementary Figure 5. Genome-wide localization of ASXL2. (**a**) Anti-AML1-ETO, RUNX1, and ASXL2 ChIP-Seq enrichment (displayed as read counts per million mapped reads) at four representative loci in SKNO-1 cells. **(b)** Distribution of ASXL2 ChIP-seq peaks across genomic regions in SKNO-1 cells (ncRNA: non-coding RNA; TTS: transcription termination sites; UTR: untranslated regions). **(c)** Histogram of ASXL2 enrichment at transcription start sites (TSS; top) and gene bodies (bottom; data displayed as read counts per million mapped reads). (**d**) Motif enrichment analysis of ASXL2 binding sites in SKNO-1 cells.

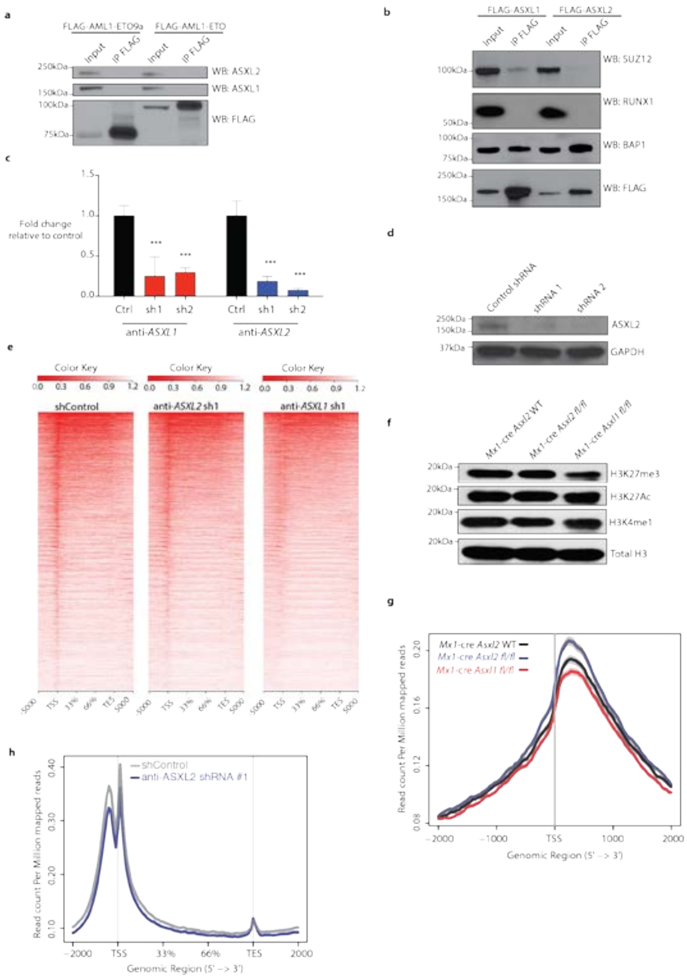

Supplementary Figure 6. ASXL2 knockdown and effects of ASXL2 loss on histone post-translational modifications. (a) Anti-FLAG immunoprecipitation of 293T cells transfected with FLAG-tagged AML1-ETO9a and AML1-ETO cDNA constructs followed by Western blotting for ASXL2, ASXL1, or FLAG proteins. **(b)** Anti-FLAG immunoprecipitation of 293T cells transfected with FLAG-tagged ASXL1 and ASXL2 cDNA constructs followed by Western blotting for SUZ12, RUNX1, BAP1, and FLAG proteins. (**c**) qRT-PCR and (**d**) Western blot for ASXL2 in SKNO-1 cells treated with control or 2 different anti-*ASXL2* shRNAs. (**e**) Heatmap representation of H3K27me3 density at transcription start sites (TSS) $\pm$ 5 kB in SKNO-1 cells with shRNA-mediated knockdown of ASXL2 or ASXL1. TES; transcription end site. **(f)** Western blot of whole-cell protein lysates of bone marrow (BM) mononuclear cells from *Mx1*-cre *Asxl2* wildtype (WT), *Mx1*-cre *Asxl2fl/fl*, and *Mx1*-cre *Asxl1fl/fl* mice. (**g**) Mean H3K27me3 ChIP-seq signal density from TSS to $\pm$ 2kB in c-Kit$^+$ BM cells from 6-week old *Mx1*-cre *Asxl2* WT, *Mx1*-cre *Asxl2fl/fl*, and *Mx1*-cre *Asxl1fl/fl* mice (cells were collected 4-weeks post-pIpC administration). (**h**) Mean H3K4me3 ChIP-seq signal density from TSSs to TESs in the SKNO-1 cells from (c) (displayed as read counts per million mapped reads). Error bars represent mean ± SD. ****p< 0.0001; p-values calculated by ordinary one-way ANOVA test.

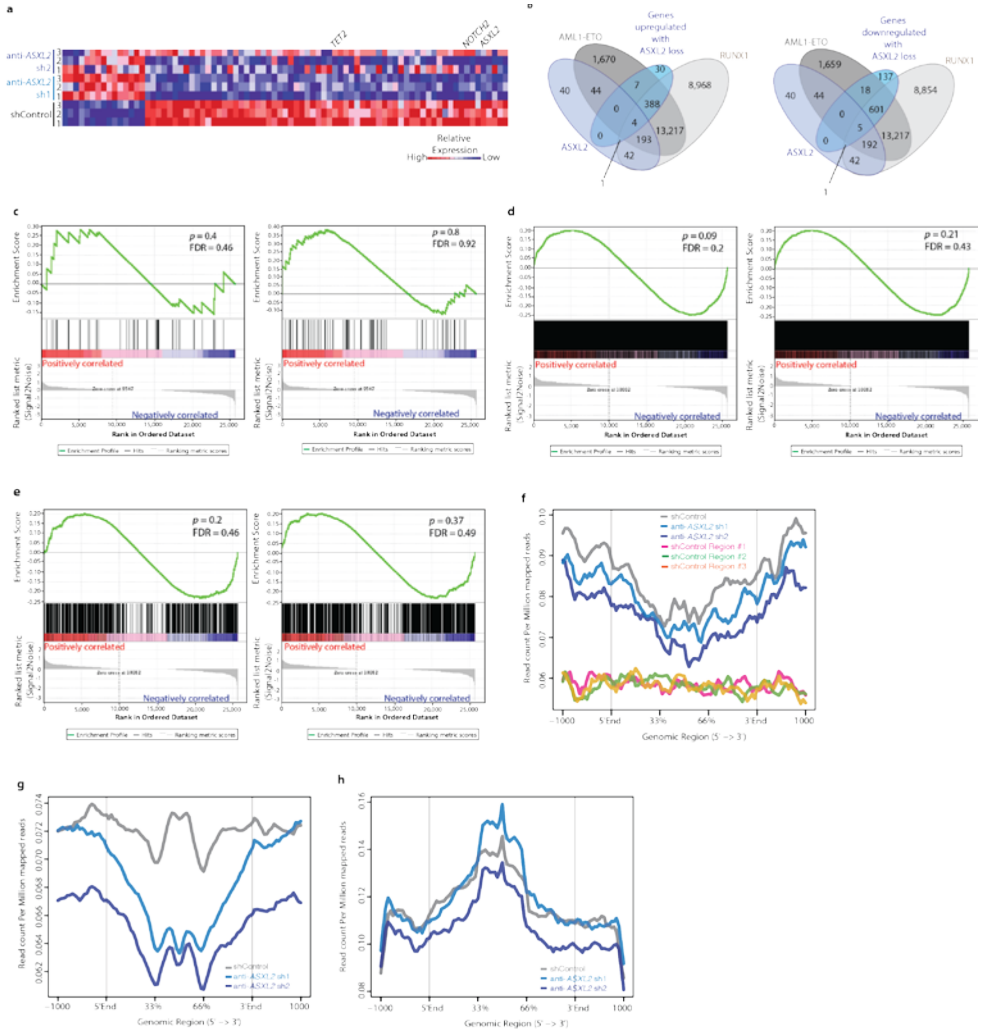

Supplementary Figure 7. Effects of ASXL2 loss on gene expression in AML1-ETO expressing acute myeloid leukemia cells and overlap with RUNX1 and AML1-ETO target genes. (a) Heatmap of genes significantly differentially expressed (*p*-value adjusted for multiple comparisons $p<0.05$) following anti-ASXL2 shRNA knockdown relative to shRNA control ("shControl") in SKNO-1 cells (3 biological replicates for each shRNA was utilized for RNA-sequencing analysis and are shown). **(b)** Overlap of genes upregulated (left) or downregulated (right) by ASXL2 loss and anti-AML1-ETO, RUNX1, or ASXL2 targets from ChIP-seq of each protein in SKNO-1 cells. **(c)** Gene set enrichment analysis (GSEA) of the effect of ASXL2 loss using one of two different anti-*ASXL2* shRNAs on the expression of genes marked by H3K27me3 in shControl cells (anti-*ASXL2* sh2 and anti-*ASXL2* sh3 cells shown on left and right respectively). GSEA of the effect of ASXL2 loss using one of two different anti-*ASXL2* shRNAs on the expression of genes within 100kB of **(d)** H3K4me1 or **(e)** H3K27Ac peaks in shControl cells (anti-*ASXL2* sh2 and anti-*ASXL2* sh3 cells shown on left and right respectively). H3K27me3 profiles at **(f)** ASXL2 or **(g)** AML1-ETO peaks in SKNO-1 cells with or without ASXL2 loss. In (f), H3K27me3 distribution at 3 random control regions of the genome outside of ASXL2 peaks is also shown. **(h)** H3K4me1 levels with or without ASXL2 loss at ASXL2 peaks in SKNO-1 cells.

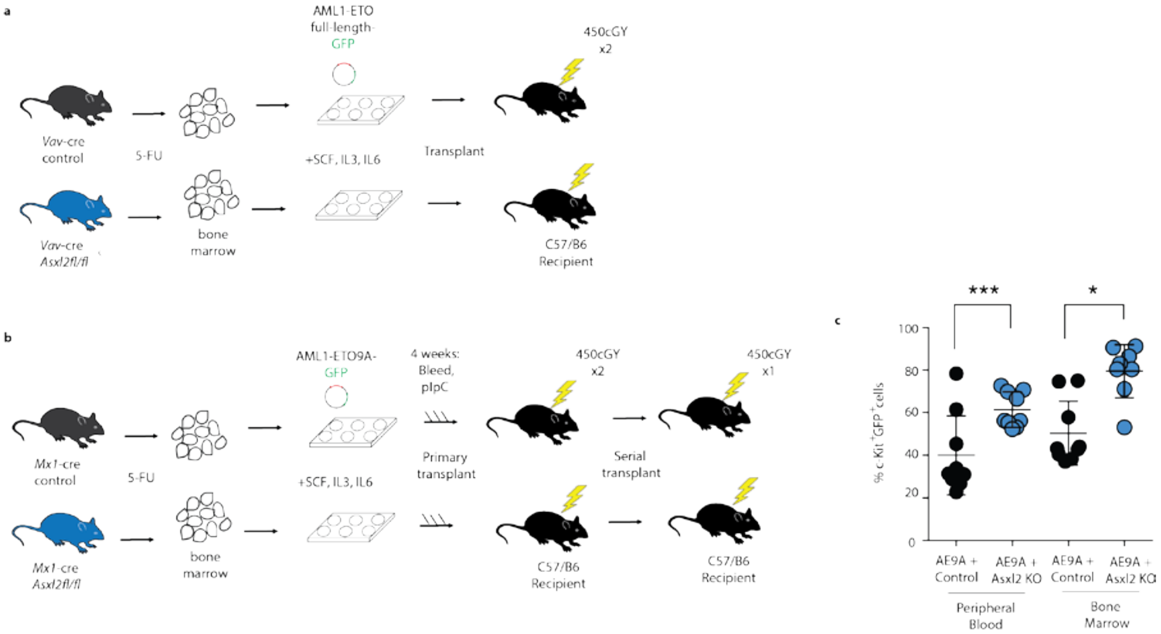

Supplementary Figure 8. Effect of AML1-ETO fusion overexpression in *Asxl2*-null hematopoietic cells. (a-b) Schema of retroviral bone marrow (BM) transplantation assay with overexpression of (a) AML1-ETO full-length or (b) AML1-ETO9a (AE9a) cDNA in Asxl2 knockout (KO) BM cells followed by serial transplantation. (**c**) Percentage of peripheral blood and BM c-Kit$^+$/GFP$^+$ double-positive cells in AE9a overexpression in control or Asxl2-deficient background at the time of sacrifice. Error bars represent mean ± SD. *$p < 0.05$, ***$p < 0.001$; p-values calculated by ordinary one-way ANOVA test.

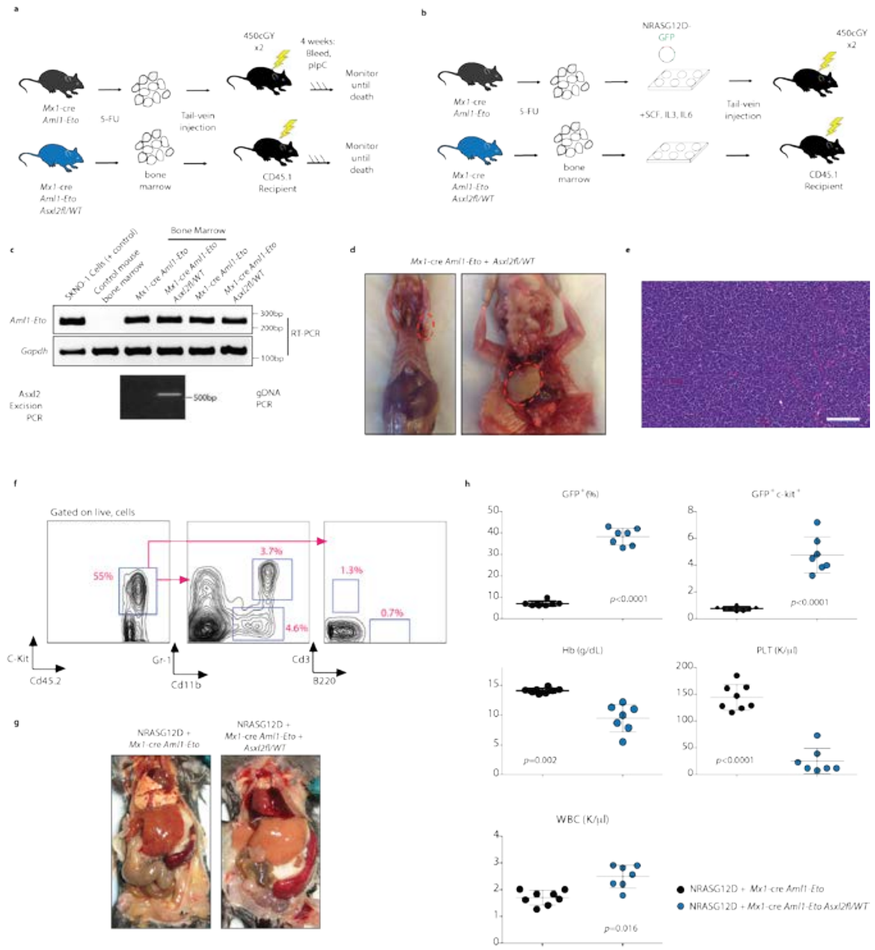

Supplementary Figure 9. Loss of Asxl2 in combination with endogenous Aml1-Eto expression results in leukemogenesis. (**a**) Schema of non-competitive transplantation assays of CD45.2 *Mx1*-cre *Aml1-Eto Asxl2* wildtype (WT) and *Mx1*-cre *Aml1-Eto Asxl2fl/WT* bone marrow (BM) cells into CD45.1 recipient mice. (**b**) Schema of retroviral overexpression of NRASG12D in CD45.2 *Mx1*-cre *Aml1-Eto Asxl2* WT or *Mx1*-cre *Aml1-Eto Asxl2fl/WT* BM cells followed by noncompetitive transplantation into CD45.1 recipient mice. (**c**) RT-PCR analysis confirming expression of *Aml1-Eto* transcript in recipients of *Mx1*-cre *Aml1-Eto Asxl2* WT or *Mx1*-cre *Aml1-Eto Asxl2fl/WT* BM cells (top) as well as genomic DNA PCR revealing excision of *Asxl2* in recipients of *Mx1*-cre *Aml1-Eto Asxl2fl/WT* BM cells (bottom). (**d**) Extramedullary leukemia (red box) in representative recipients of *Mx1*-cre *Aml1-Eto Asxl2fl/WT* BM cells. (**e**) Histologic and (**f**) FACS analysis of extramedullary leukemia from mouse in (d) revealing acute myeloid leukemia. (**g**) Photographs of liver and spleen of recipients of NRASG12D + *Mx1*-cre *Aml1-Eto* or NRASG12D + *Mx1*-cre *Aml1-Eto* + *Asxl2fl/wt* BM cells at time of sacrifice. (**h**) Percentage of peripheral blood GFP^{+} and GFP^{+}/c-Kit^{+} double-positive cells as well as hemoglobin (Hb), platelet, and white blood cell (WBC) counts in recipients of NRASG12D + *Mx1*-cre *Aml1-Eto* or NRASG12D + *Mx1*-cre *Aml1-Eto* + *Asxl2*fl/wt BM cells at the time of sacrifice. Error bars represent mean ± SD. *p*-values calculated by ordinary one-way ANOVA test.

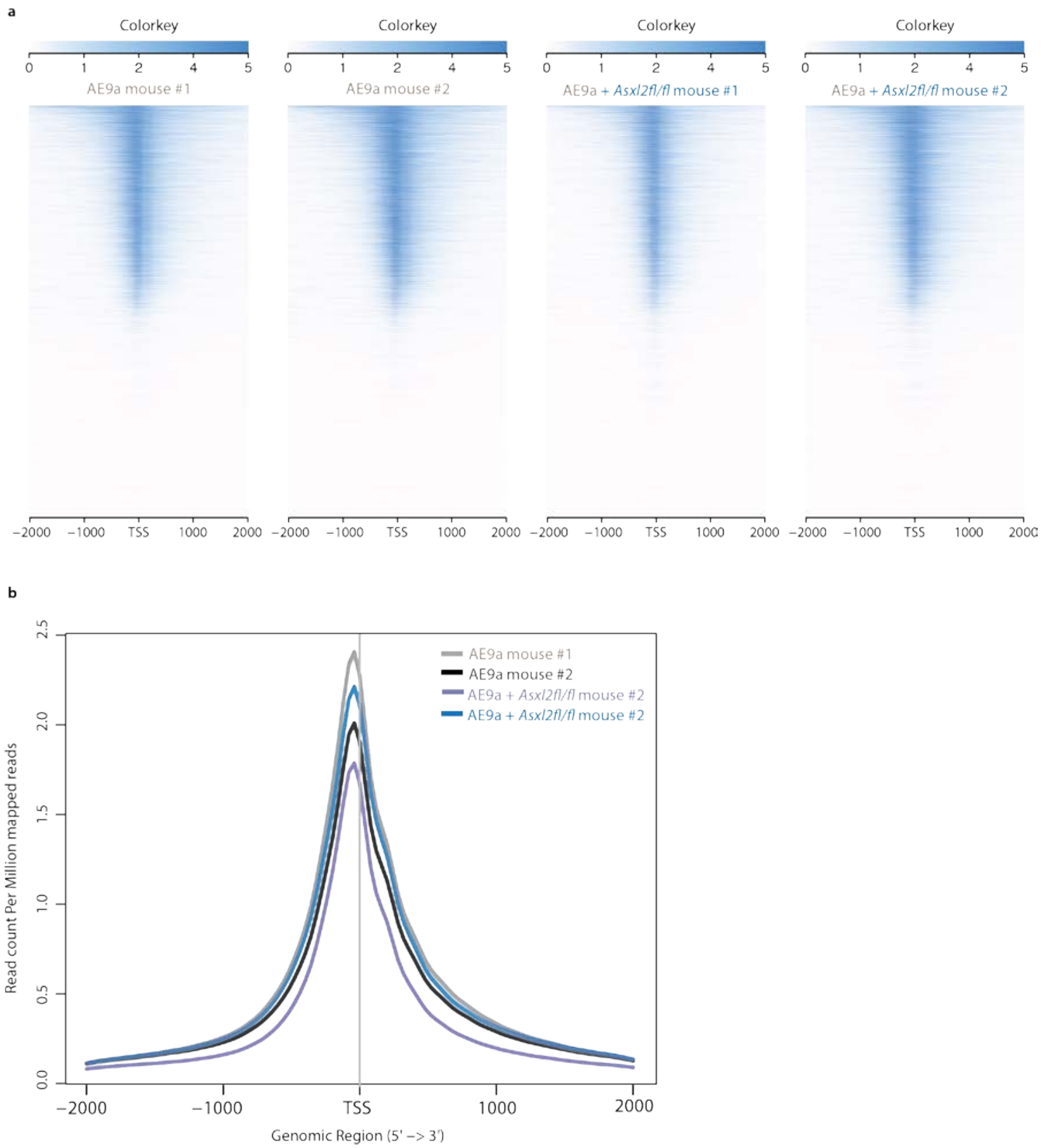

Supplementary Figure 10. Global chromatin accessibility profiles of AML1-ETO9a (AE9a) acute myeloid leukemias with or without Asxl2 loss as assessed by assays of transposase-accessible chromatin sequencing (ATAC-seq). Transposase hypersensitive site (THS) density was analyzed genome wide and found in promoter regions (±2 kb of annotated RefSeq transcription start sites (TSS)) as displayed by **(a)** heatmap distribution of THSs or **(b)** histogram (data displayed as read counts per million mapped reads).

Supplementary Table 1. qRT-PCR primers used in this study.

Human

Gene	Forward	Reverse
hASXL1	GAAAAGCCACAGCCCACTAA	CAGAGCACGGGCTTTAAT
hASXL2	AATACCCCAATACACCCATGA	TCCCATTCTACCTGGAACCTT
hGAPDH	GGAGCGAGATCCCTCCAAAAT	GGCTGTTGTCATACTTCTCATGG

Mouse

Gene	Forward	Reverse
mAsxl1	CAGCCGTTTTACCACAGTTT	AGGGAAAGGGACAGAATGAC
mAsxl2	AGCTGGGAGAAAAGACCACG	CTTGGACCCTGTCCCCTCTA
mAsxl3	ACATTGGATCTAGTCGTTGACCC	CGTACACACCCTGTTCTCTTCTC
mActin	GATCTGGCACCACACCTTCT	CCATCACAATGCCTGTGGTA
